# Exploratory study of a multifrequency EIT-based method for detecting intracranial abnormalities

**DOI:** 10.3389/fneur.2023.1210991

**Published:** 2023-08-11

**Authors:** Jieshi Ma, Jie Guo, Yang Li, Zheng Wang, Yunpeng Dong, Jianxing Ma, Yan Zhu, Guan Wu, Liang Yi, Xuetao Shi

**Affiliations:** ^1^Department of Medical Engineering, Army Medical Center of PLA, Chongqing, China; ^2^Institute of Medical Research, Northwestern Polytechnical University, Xi'an, China; ^3^Department of Neurosurgery, Army Medical Center of PLA, Chongqing, China; ^4^Hangzhou Utron Technology Co., Ltd., Hangzhou, China; ^5^Department of Medical Electronic Engineering, School of Biomedical Engineering, Air Force Medical University of PLA, Xi'an, China

**Keywords:** cerebral, MFEIT, image features, area and signal intensity of ROI, symmetry, intracranial abnormality, detection

## Abstract

**Objective:**

The purpose of this paper is to compare the differences in the features of multifrequency electrical impedance tomography (MFEIT) images of human heads between healthy subjects and patients with brain diseases and to explore the possibility of applying MFEIT to intracranial abnormality detection.

**Methods:**

Sixteen healthy volunteers and 8 patients with brain diseases were recruited as subjects, and the cerebral MFEIT data of 9 frequencies in the range of 21 kHz - 100 kHz of all subjects were acquired with an MFEIT system. MFEIT image sequences were obtained according to certain imaging algorithms, and the area ratio of the ROI (AR_ROI) and the mean value of the reconstructed resistivity change of the ROI (MVRRC_ROI) on both the left and right sides of these images were extracted. The geometric asymmetry index (GAI) and intensity asymmetry index (IAI) were further proposed to characterize the symmetry of MFEIT images based on the extracted indices and to statistically compare and analyze the differences between the two groups of subjects on MFEIT images.

**Results:**

There were no significant differences in either the AR_ROI or the MVRRC_ROI between the two sides of the brains of healthy volunteers (*p* > 0.05); some of the MFEIT images mainly in the range of 30 kHz – 60 kHz of patients with brain diseases showed stronger resistivity distributions (larger area or stronger signal) that were approximately symmetric with the location of the lesions. However, statistical analysis showed that the AR_ROI and the MVRRC_ROI on the healthy sides of MFEIT images of patients with unilateral brain disease were not significantly different from those on the affected side (*p* > 0.05). The GAI and IAI were higher in all patients with brain diseases than in healthy volunteers except for 80 kHz (*p* < 0.05).

**Conclusion:**

There were significant differences in the geometric symmetry and the signal intensity symmetry of the reconstructed targets in the MFEIT images between healthy volunteers and patients with brain diseases, and the above findings provide a reference for the rapid detection of intracranial abnormalities using MFEIT images and may provide a basis for further exploration of MFEIT for the detection of brain diseases.

## 1. Introduction

Most brain diseases have extremely high rates of death and disability (1, 2). Some brain diseases, such as stroke, have an abrupt onset and rapid progression. There are some advanced diagnostic techniques, such as computed tomography (CT) and magnetic resonance imaging (MRI). However, CT and MRI cannot meet some urgent clinical needs, such as the monitoring and early detection of an intraoperative brain injury, real-time monitoring and assessment of intracranial conditions of patients with critical brain diseases, or dynamic evaluation of the effects of drugs for cerebral edema treatment, due to their large size, high costs and risk of radiation exposure. Therefore, an imaging technology that is portable has high sensitivity in detecting intracranial conditions and can dynamically and continuously acquire brain imaging data from patients at the bedside in real time is urgently needed to help doctors make early diagnoses and take timely measures to not only save patients’ lives but also greatly improve the prognosis of patients with brain diseases.

The special anatomy of the brain makes it a world-class challenge to investigate technologies suitable for the detection and continuous monitoring of brain diseases. However, current imaging technologies are deficient in terms of practical applications. For example, although ultrasound imaging techniques can theoretically reflect the occurrence of brain diseases, ultrasound cannot penetrate the skull, and ultrasonography is only applicable for infants with unclosed skulls (3), while for adults, the ultrasound probe can only be placed on the skull bone suture to detect nearby intracranial conditions, and this technique can only detect a limited range of brain lesions. In addition, NIR spectroscopic imaging techniques can be used to identify cerebral lesions by applying certain wavelengths of NIR light to the surface of the human head and using the abnormal intensity of reflection according to the different absorption rates of NIR light by different brain tissues. However, the NIR optical signal attenuates with increasing depth into the skull (4) and generally has good detection ability for tissues within 1–2 cm under the skull (5); the deepest detection range is up to 4 cm below the skull (6), which is not suitable to the detection of deep brain lesions.

Electrical impedance tomography (EIT) is a novel medical imaging technique that is based on the principle of applying a certain safe excitation current to the human surface while measuring the body surface voltage and then reconstructing the image according to a specific imaging algorithm. Moreover, EIT is advantageous in that it is noninvasive, used in functional imaging, does not use radiation, and uses a small, portable device, which has great potential and application in the rapid detection of intracranial conditions in patients with brain diseases. In 2013, a researcher reported the feasibility of using EIT to monitor intracranial electrical impedance changes in humans (7). The EIT technique in this report is time-differential EIT (tdEIT), which reflects the change in the resistivity distribution of the measured cranial brain at a point in time relative to the resistivity distribution at a reference point in time. This algorithm is fast and greatly reduces systematic and measurement errors in the data by the time difference. However, the shortcoming of tdEIT is that in practice, it is difficult to obtain reference data corresponding to the time before disease onset, so it is not suitable for situations where lesions have formed and do not change over a short time. Therefore, there is a need for a one-time brain EIT imaging technique that can reflect the absolute intracranial condition and examine the intracranial promptly during the patient monitoring period so that the progression of brain disease can be assessed dynamically.

It has been found that static EIT, symmetric imaging and multi-frequency EIT(MFEIT) techniques are currently suitable for one-time cerebral EIT imaging. The static EIT technique reconstructs the absolute value of the resistivity distribution within the subject and may provide more complete and reliable information, but the impact of its model error and measurement noise on the quality of the reconstructed image is particularly severe. As a result, the static imaging algorithm has extremely strict requirements for data quality and electrode position, which, together with the long imaging time of this algorithm, is highly limited in clinical applications. Most of the current research on this technology is focused on breast and lung imaging, mainly for optimization of imaging quality and improvement of imaging speed, basically at the stage of numerical simulation studies and physical model studies (8–10), and individual studies have imaged the human chest and lungs (11, 12). Due to the complex anatomy of the human head and the high resistivity of the skull, the imaging results of the static EIT technique in the human brain have not been reported.

Symmetrical imaging is an EIT imaging technique designed in a specific application scenario of the human brain in which it is assumed that the resistivity distribution of the healthy human brain is approximately left–right symmetric, that brain lesions are usually located unilaterally or that bilateral lesions of the brain cannot be completely symmetric and that lesions will break the original symmetry of the resistivity distribution of the healthy human brain. Based on this assumption, in 2014, an investigator proposed a symmetrical EIT (Symmetrical EIT, SEIT) method, which implemented imaging of simulated brain lesions on an ideally symmetric brain simulation model and a physical model of the brain (13), but the imaging results of this method on the human brain have not been reported because its assumption only holds on the human brain with inherently good impedance symmetry. In 2018, a similar symmetry imaging method called “SdEIT” was reported, and its feasibility was verified on simulation and physical models (14). In 2019, a dual-frequency SdEIT method was reported, and its application scope and requirements were studied based on the measured data of patients with brain diseases. The results showed that the method had high requirements for the impedance symmetry of the human brain and the symmetry of the electrode position (15), and the conditions for its practical application are demanding. The asymmetry of the human brain itself and the asymmetry of the measurements limit the practical application of symmetric electrical impedance imaging, and no imaging results of such methods on bulk human brains have been seen.

MFEIT, also named “frequency-difference EIT (fdEIT),” reflects the variation in the resistivity distribution inside the subject with frequency based on the differential components of the EIT data at two different frequency points of the subject, and similar to temporal differential imaging, the frequency differential can greatly reduce the influence of systematic and measurement errors in the data. In addition, because there are large differences in the impedance spectra between normal human brain tissues and diseased brain tissue in specific frequency bands, it is possible to reflect the intracranial condition (whether it has occurred and the location of the lesion, etc.) of patients with brain diseases by MFEIT in specific frequency bands. In 2006, the UK UCL research team reported an MFEIT system for detecting brain lesions (16) and obtained the frequency difference imaging results of simulated brain lesions on the human brain physical model. In 2012, the group compared the results of five fdEIT imaging algorithms on a phantom of the human brain and hypothesized that the weighted fdEIT is expected to be used for imaging lesions in the human brain (17). In 2014, the group proposed a spectrally constrained MFEIT algorithm for differentiating the types of stroke lesions and imaged them on a simulation model of the human brain, but the imaging results were easily affected by electrode errors and errors in the shape of measured objects (18). In 2016, the group reduced the impact of electrode model error on the imaging quality of multifrequency EIT by reconstructing the conductivity spectrum and the positions of electrodes simultaneously (19). In the same year, another group reported a spectral decomposition fdEIT algorithm and imaged simulated stroke lesions on a phantom of the human brain (20). However, the imaging results of MFEIT in the human brain have not yet been reported, and the differences in cerebral MFEIT images between healthy individuals and patients with brain diseases have not been reported, which limits the application of MFEIT in imaging the human brain.

In this study, cerebral MFEIT data from a group of healthy volunteers and a group of patients with brain diseases were collected and subjected to frequency-difference imaging, and the symmetry of cerebral MFEIT images was analyzed to summarize the differences in the characteristics of those images between healthy individuals and patients with brain diseases, laying the foundation for the establishment of a rapid assessment method for intracranial abnormalities based on cerebral MFEIT images.

## 2. Methods

### 2.1. Experimental subjects

Two groups of people were recruited to participate in the study. The healthy group included 16 individuals (male, age 40.25 ± 11.18). No history of brain disease and no abnormalities were seen on plain CT scans of their brains. The patient group included 8 patients (P1 – P8) with brain diseases (7 males, age 59 ± 10.46). Obvious lesions were visible on the patients’ CT or MRI images, which represented 6 patients with intracranial hemorrhage, 1 patient with cerebral ischemia, and 1 patient with cerebral edema. The specific information of the patients is shown in Table 1. The clinical trial conducted in this study was approved by the Ethics Committee of the Army Medical Center of PLA with consent for implementation. All volunteers were informed of the purpose and content of the experiment and signed the informed consent form before participating in the experiment.

**Table 1:** Specific information of patients.

NO.	Sex	Age	Imaging diagnosis	Time from onset of brain diseases to EIT data acquisition
P1	Male	72	Cerebral hemorrhage in right frontal lobe	6d 3.8 h
P2	Male	48	Hemorrhage in right outer capsule area and basal ganglia area	9.2 h
P3	Male	75	Cerebral contusion and laceration of left frontotemporal lobe, subdural hematoma of left frontotemporal lobe, extradural hematoma of left frontotemporal lobe	2d 3.1 h
P4	Male	59	Left frontal lobe hemorrhage	5d 21.7 h
P5	Male	64	Cerebral infarction in right hemisphere	2d 16.4 h
P6	Female	48	Left frontal lobe edema	1d 23.3 h
P7	Male	52	Right cerebellar hemisphere hemorrhage	1d 16.1 h
P8	Male	54	Subdural hematoma of right frontal lobe, contusion and laceration of left temporal lobe	10d 3.5 h

### 2.2. Experimental procedures

#### 2.2.1. Data acquisition

**Figure 1:**
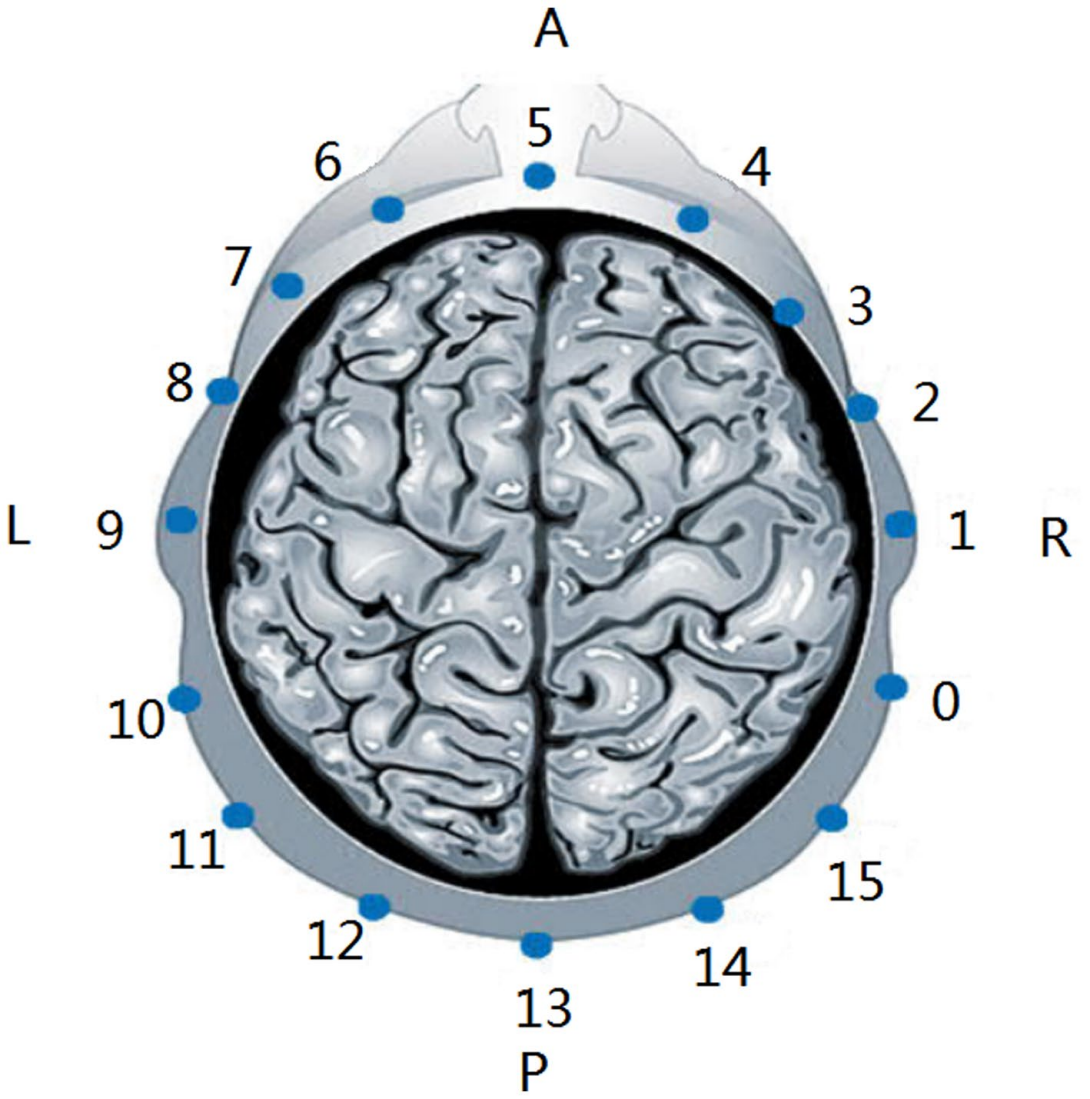
Locations of the cerebral EIT electrodes. A, anterior. P, posterior. L, left. R, right.

Data acquisition was performed using a cerebral MFEIT named EH-300 (Hangzhou Utron Technology Co., Ltd.). The system uses 16 electrodes applied to the subject’s scalp to refer to the locations of the cerebral EIT electrodes, i.e., 16 electrode locations equally spaced on the intersection line of the two planes, one of which was through three points located at 1 cm above the eyebrows and 1 cm above the left and right ears, and the other plane was the cranial surface, as shown in Figure 1. MFEIT data were acquired at nine frequencies (i.e., a frequency cycle), including 21 kHz, 30 kHz, 40 kHz, 0.90 kHz, and 100 kHz. The driving current applied to the subjects’ heads was a sinusoidal alternating current with a root mean square value of 176 μA. The opposite excitation-adjacent measurement mode was adopted to decide the electrode pair (e.g., 5–13) excited by the current and the corresponding electrode pairs (e.g., 6–7, 7–8, 8–9, 9–10, 10–11, 11–12, 14–15, 15–0, 0–1, 1–2, 2–3, 3–4) to measure the boundary voltages that form the EIT data after the current excites all the opposite electrode pairs.

**Figure 2:**
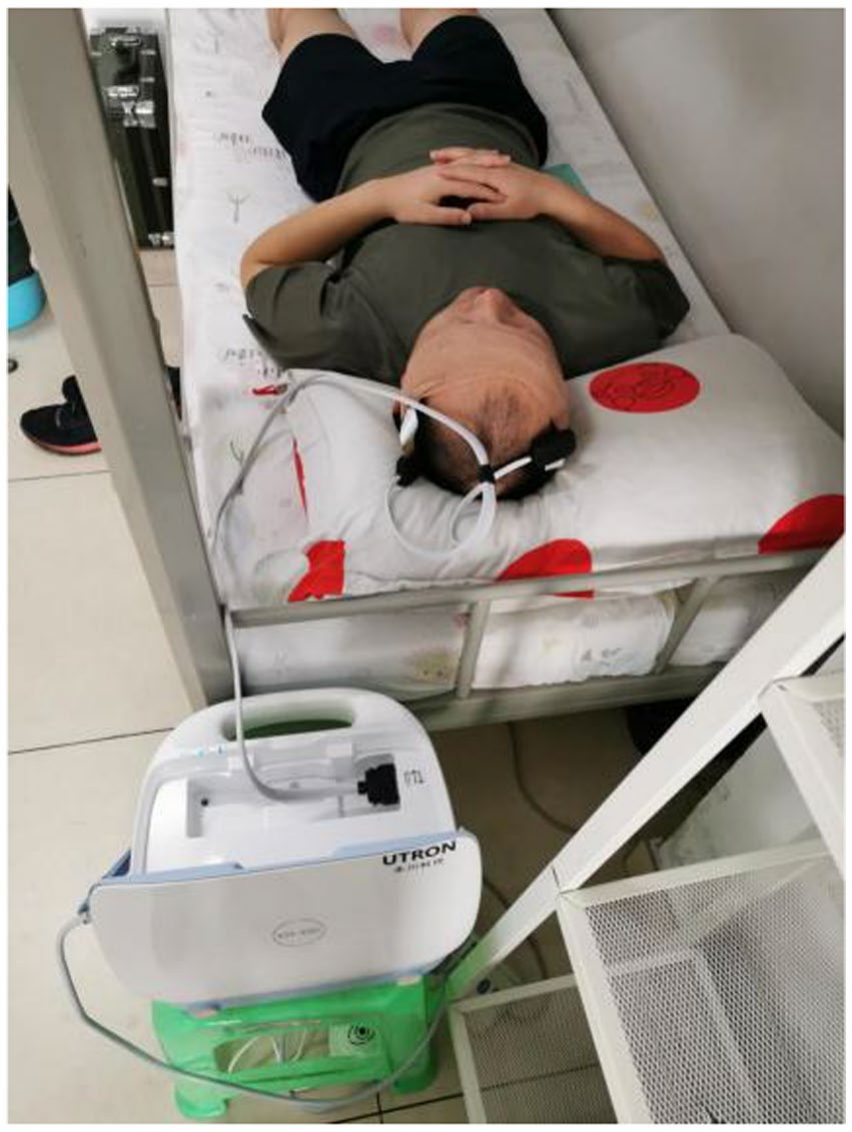
Photographs of MFEIT data acquisition in the healthy volunteers.

Cerebral EIT data collection from healthy volunteers was performed in a dedicated laboratory with temperature controlled at 24 ± 1°C and 52% ± 1% humidity in 2022. The subjects were lying in the horizontal position with 16 electrodes placed equidistantly on their scalp, and then the EH-300 was used to detect the electrode contact impedance to determine whether the electrodes were making good contact, and the electrodes with poor contact were adjusted until the contact impedance of all electrodes was below the limit of 350 ohms. After the electrodes were applied, the MFEIT data of the subjects’ brains were collected (as shown in Figure 2). Two frequency cycles were acquired.

**Figure 3:**
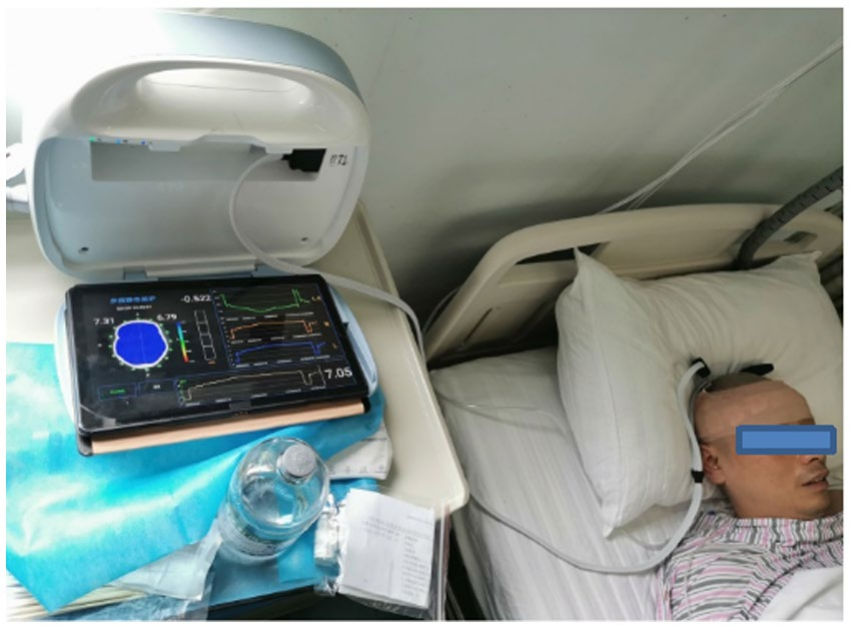
Photograph of MFEIT data acquisition in patients with brain diseases in the neurosurgical intensive care unit.

Data from patients with brain diseases were collected at the neurosurgical intensive care unit at the Army Medical Center of PLA in 2022. The cerebral EIT data of the patients were collected in the same position as the healthy individuals. The parameter settings of the EIT system and data collection requirements were the same as those for healthy volunteers. The experimental photograph of the cerebral EIT data acquisition in the patients is shown in Figure 3.

#### 2.2.2. Data processing and calibration

**Figure 4:**
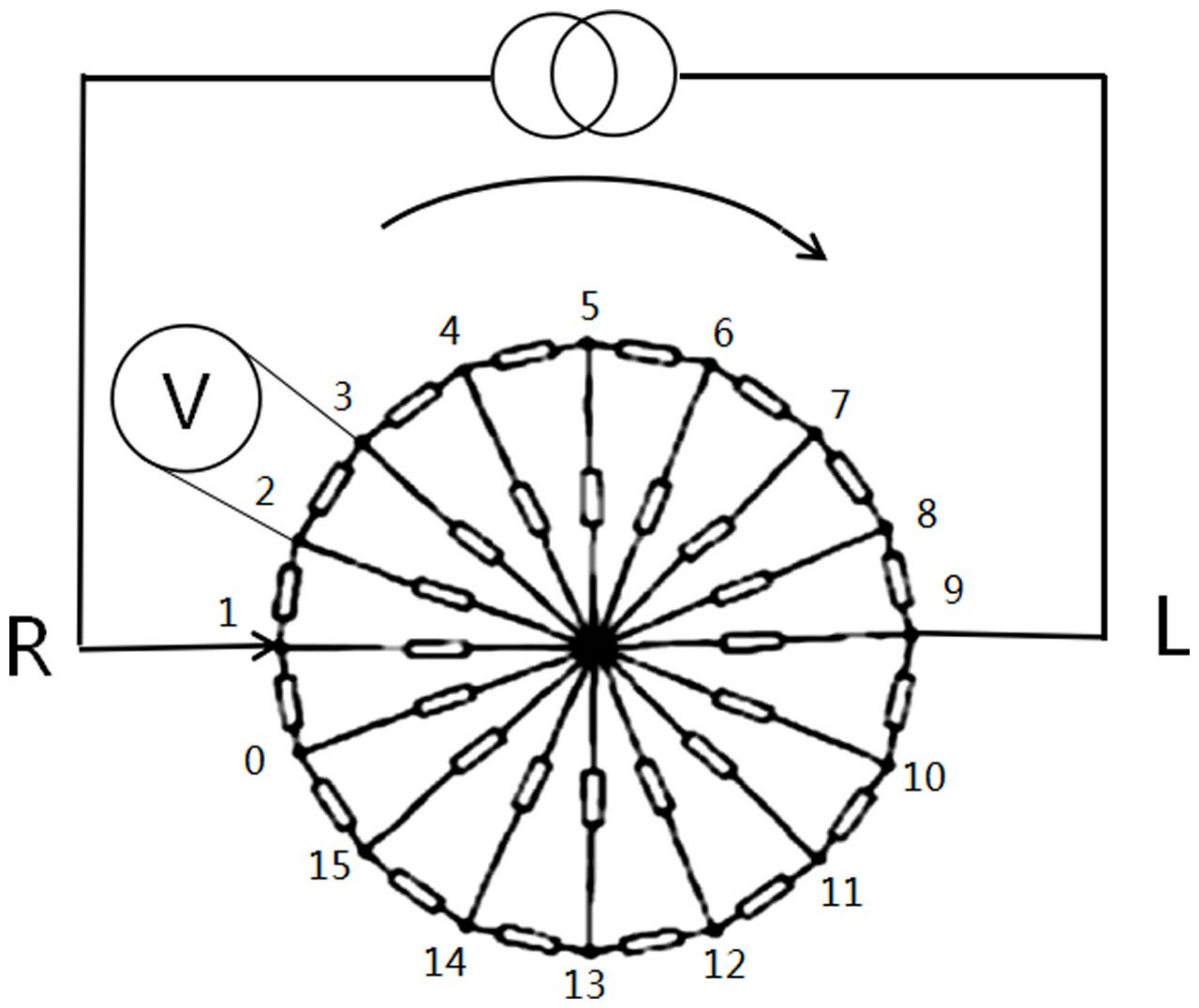
Circuit diagram of the calibration PCB used to calibrate the MFEIT data of the human brain for real measurements.

The consistency of the frequency response characteristics of EIT measurements should be ensured; thus, we calibrated the EIT acquisition device before performing fdEIT imaging. The calibration is based on the measurements of the acquisition device EH-300 on a printed circuit board (PCB). The calibration PCB is composed of thirty-two 499-ohm precision resistors in a hub-and-spoke structure, as shown in Figure 4. Before calibration, the MFEIT data of the calibration PCB were collected as standard data. The human data were then collected as the data to be calibrated before imaging, and the ratio of the theoretical transmission impedance of the calibration PCB at a certain frequency to the measured EIT boundary data of the calibration PCB at that frequency was used as the amplification factor A. The calibrated human brain data were then obtained by multiplying the MFEIT data to be calibrated by the corresponding amplification factor A.

### 2.3. MFEIT imaging and image feature analysis

The MFEIT reconstruction algorithm uses the frequency-difference damping least squares method with standard Tikhonov regularization (STR) (21) to select the EIT data at 21 kHz as the background frame and the other 8 higher frequency points as the foreground frames and to image the changes in resistivity distribution corresponding to the boundary voltage changes of a foreground frame relative to the background frame in a circular imaging domain. The upper side of the EIT image corresponds to the forehead of the human brain, the lower side of the EIT image corresponds to the occipital region of the human brain, the left side of the EIT image corresponds to the right side of the human brain, and the right side of the EIT image corresponds to the left side of the human brain. These directions are consistent with the directions of the brain CT images. On the reconstructed EIT image, a red pixel represents a decrease in resistivity at the location, while a blue pixel represents an increase in resistivity at the location. Moreover, a green pixel on the EIT image represents a very small change (increase or decrease) in resistivity at the location. There is a color scale bar on the right beside the EIT image. A certain color scale corresponds to a reconstructed resistivity change value. The five decimals on the right beside the color scale bar are the +Amax, +0.5Amax, 0.000, −0.5Amax and -Amax, where Amax is the maximum absolute value of the reconstructed resistivity change values of the whole EIT image. On the reconstructed EIT image, some closed areas can be recognized by the human eye, which are composed of pixels with higher reconstructed resistivity values. These areas are called reconstructed targets.

#### 2.3.1. Extraction of effective regions of interest

First, the pixels within the reconstructed targets on the cerebral MFEIT image with the absolute resistivity change values in the range of 62.5%Amax - 100%Amax are identified as composing the region of interest (ROI). The ROI in contact with the imaging boundary, the ROI crossed by the vertical symmetry axis of the imaging domain and the small target area (the ROI cut by the vertical symmetry axis into two areas, each with a pixel (or area) ratio less than 0.85% between the area on each side and the overall imaging domain is the small target) are excluded because they have reconstruction errors or are not valuable for analysis of the symmetry of the cerebral MFEIT images, and the remaining ROIs are the effective ROIs.

#### 2.3.2. Characterization of MFEIT images

Based on the extracted effective ROIs, the differences in the area of the reconstructed targets and their signal intensities between the left and right sides of healthy individuals and between the normal and affected sides of patients with brain diseases could be analyzed.

The area of the reconstructed targets on one side of the MFEIT image was measured by the area ratio of the ROIs (AR_ROI) and the ROIs were on the corresponding side within the reconstructed targets. The AR_ROI of the side was calculated by extracting the number of pixels in the effective ROIs on the corresponding side of each fdEIT image for each subject, and if there were multiple effective ROIs on a single side, the total number of pixels of those ROIs was taken as the area of the ROIs on that side, and then the total number of pixels on the left and right sides was divided by the total number of pixels in the circular imaging domain to obtain the AR_ROI on the left and right sides of the measured subject. Special cases that might be encountered in the analysis were handled as follows: if no effective ROI was found on one side, the area ratio of the target area on that side was considered 0.

The signal intensity of the reconstructed pixels on one side of the MFEIT image was evaluated by the mean absolute value of the reconstructed resistivity change of the ROI (MVRRC_ROI) and the ROI was within the reconstructed targets on the corresponding side and should contain the pixel with the maximum reconstructed resistivity value (absolute value) on the side of each fdEIT image. If there was no effective ROI on one side, the outline of the ROI with the maximum reconstructed resistivity change value on the opposite side was mirrored to this side according to the symmetric relationship between left and right, and the average value of the reconstructed resistivity change value of all pixels within the mirrored outline was used as the signal intensity on this side.

The area of reconstructed targets and the signal intensity of both sides were evaluated in 16 healthy volunteers, and the differences between the left and right sides were analyzed. Furthermore, for patients with unilateral lesions, the area of the reconstructed targets and their signal intensity on the left and right sides were evaluated and the differences between the affected sides and the healthy sides were analyzed.

Considering that a salient feature of the cerebral MFEIT images of the healthy volunteers is the approximate left–right symmetry, to further quantify the symmetry of MFEIT images, a symmetry analysis on the obtained cerebral MFEIT images of the subjects, including structural symmetry analysis and signal intensity symmetry analysis, was performed.

#### 2.3.3. Geometric symmetry analysis

The main purpose of the geometric symmetry analysis was to assess the symmetry of the positions of the effective ROIs on the left and right sides of the reconstructed EIT images. The analysis method of ROI geometric symmetry varied with the number of effective ROIs on the left and right sides of the MFEIT images, which are as follows.

In the first case, when only one side of the left and right sides of the imaging domain contained an effective ROI, we denoted the geometric symmetry in this case as “not symmetrical (NS).”

In the second case, there was one effective ROI on both sides of the imaging domain, the intersection point of the vertical symmetry axis of the imaging domain and the lower arc of the imaging circle was used as the origin to construct an XY coordinate system, based on which the distances from the barycenter of the effective ROIs to the X and Y axes on the left and right sides of the MFEIT images could be calculated, and the geometric asymmetry index (GAI) of the effective ROIs on the left and right sides of the multifrequency electrical impedance images were calculated as follows.

(1) $$GAI=0.5*\left\{\begin{array}{l} \left[2*\mathrm{abs}\left(L_{x}-R_{x}\right)/\left(L_{x}+R_{x}\right)\right]\\ +\;\;\left[2*\mathrm{abs}\left(L_{y}-R_{y}\right)/\left(L_{y}+R_{y}\right)\right] \end{array}\right\}*100\%$$

where $L_{x}$ is the distance from the barycenter of the ROI on the left side to the X-axis, $R_{x}$ is the distance from the barycenter of the ROI on the right side to the *X*-axis, $L_{y}$ is the distance from the barycenter of the ROI on the left side to the *Y*-axis, and $R_{y}$ is the distance from the barycenter of the ROI on the right side to the *Y*-axis.

In the third case, when the number of effective ROIs on one side of the imaging domain was ≥2, the following method was used. First, the geometric asymmetry index (GAI) of each effective ROI on one side and each effective ROI on the other side was derived separately according to Eq. (1), and then all geometric asymmetry indices were averaged and taken as the final geometric asymmetry index (GAI).

#### 2.3.4. Signal intensity symmetry analysis

The purpose of the signal intensity symmetry analysis was to analyze the difference in the intensity of the reconstructed signals of the effective ROIs on the left and right sides of the imaging domain. First, the effective ROI on the EIT image was determined according to the method described above, and the average of the absolute values of the reconstructed resistivity change values of all pixel points within the effective ROI on the MFEIT image was calculated as the average signal intensity of the effective ROI. The intensity asymmetry index (IAI) of an ROI and its contralateral region was calculated based on the average signal intensity of the ROI and its contralateral symmetrical region by the following equation.

(2) $$\begin{array}{c} IAI=2*\mathrm{abs}\left(I_{ROI}-I_{contralateral}\right)\\ /\left(I_{ROI}+I_{contralateral}\right)*100\% \end{array}$$

where $I_{ROI}$ is the average signal intensity of an effective ROI and $I_{contralateral}$ is the average signal intensity of the symmetric region on the opposite side of this ROI. For example, if there were a total number, n, of effective ROIs on the left and right sides (*n* ≥ 1), the intensity asymmetry index (IAI) of each effective ROI was first separately calculated using Eq. (2), and then the average of these intensity asymmetry indices was found, and the average value was taken as the final intensity asymmetry index (IAI).

#### 2.3.5. Analysis of the incidence of positive events

When the AR_ROI of the affected side on the MFEIT image of patients with unilateral brain disease was smaller than that of the healthy side or the signal intensity of the ROI on the affected side was lower than that on the healthy side, the event was considered a positive event, and conversely, it was a negative event. By counting the proportion of positive events (positive rate) among all patients at each frequency point and based on the observation of the change in positive rate with frequency, the intersection of the frequency range with positive rates>50% on both AR_ROI and SI was selected as the key frequency range for the occurrence of positive events.

#### 2.3.6. Statistical analysis

Statistical analysis was performed in R language (version 4.2.2), and since the normality and the variance homogeneity of the two groups of data to be analyzed at different frequency points were not the same, the Wilcoxon rank sum test, a nonparametric statistical analysis method, was used to analyze the differences between the left side and the right side in the area ratio of the ROI and signal intensity in healthy volunteers, to analyze the difference in the incidence between the positive event and the negative event and to analyze the differences between the normal side and the affected side in the area ratio of the ROI and signal intensity in patients with brain diseases. The same statistical method was further used to test the hypothesis of the relative highs and lows of the geometric asymmetry index and intensity asymmetry index between healthy individuals and patients with brain diseases at each frequency point. The significance levels were all set at *p* < 0.05 for the above tests.

## 3. Results

### 3.1. Imaging results

**Figure 5:**
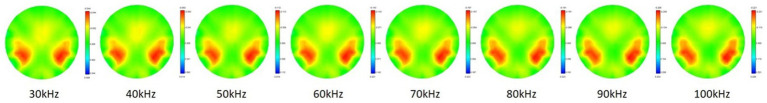
Cerebral MFEIT imaging results of a healthy volunteer (30 kHz-100 kHz).

The MFEIT images of 16 healthy volunteers were reconstructed. The cerebral MFEIT images of healthy individuals are basically left–right symmetric, and the variation in electrical impedance of the left brain with frequency was similar to that of the right brain, the MFEIT images of a healthy subject is shown in Figure 5, while images of the other 15 individuals are attached in the Appendix. Moreover, two stronger resistivity distributions with approximate left–right symmetry appeared in the posterior part of each healthy brain.

**Figure 6:**
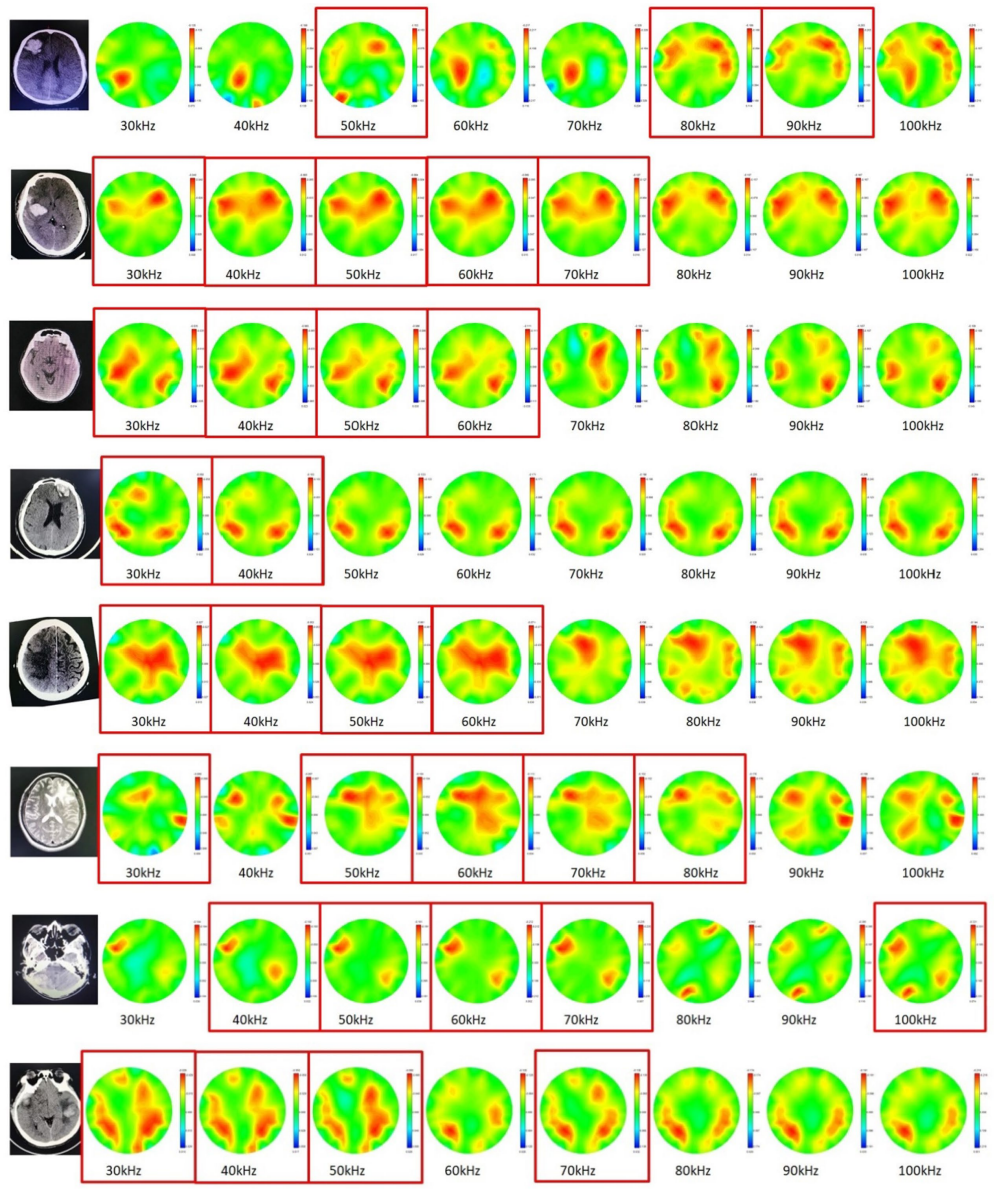
Cerebral MFEIT imaging results of the patients (P1 – P8) with brain diseases (30 kHz-100 kHz). The red box corresponds to the observed high electrical resistivity changes in the symmetrical area on the opposite side of the patient’s lesion.

The results of cerebral MFEIT imaging of the eight patients (P1 - P8) with brain diseases are shown in Figure 6, one patient per row, with the following characteristics: the asymmetry of MFEIT images was more pronounced in patients with brain diseases than in healthy subjects; an area of stronger resistivity distribution was presented in the contralateral near-symmetric position of the lesion area, or the intensity (or area) of the reconstructed resistivity distribution of the lesion area was lower than that of the symmetric area on the unaffected side. The corresponding images are marked by the red solid box.

### 3.2. Analysis of the difference in the area ratio and signal intensity of the ROIs between the two sides

**Figure 7:**
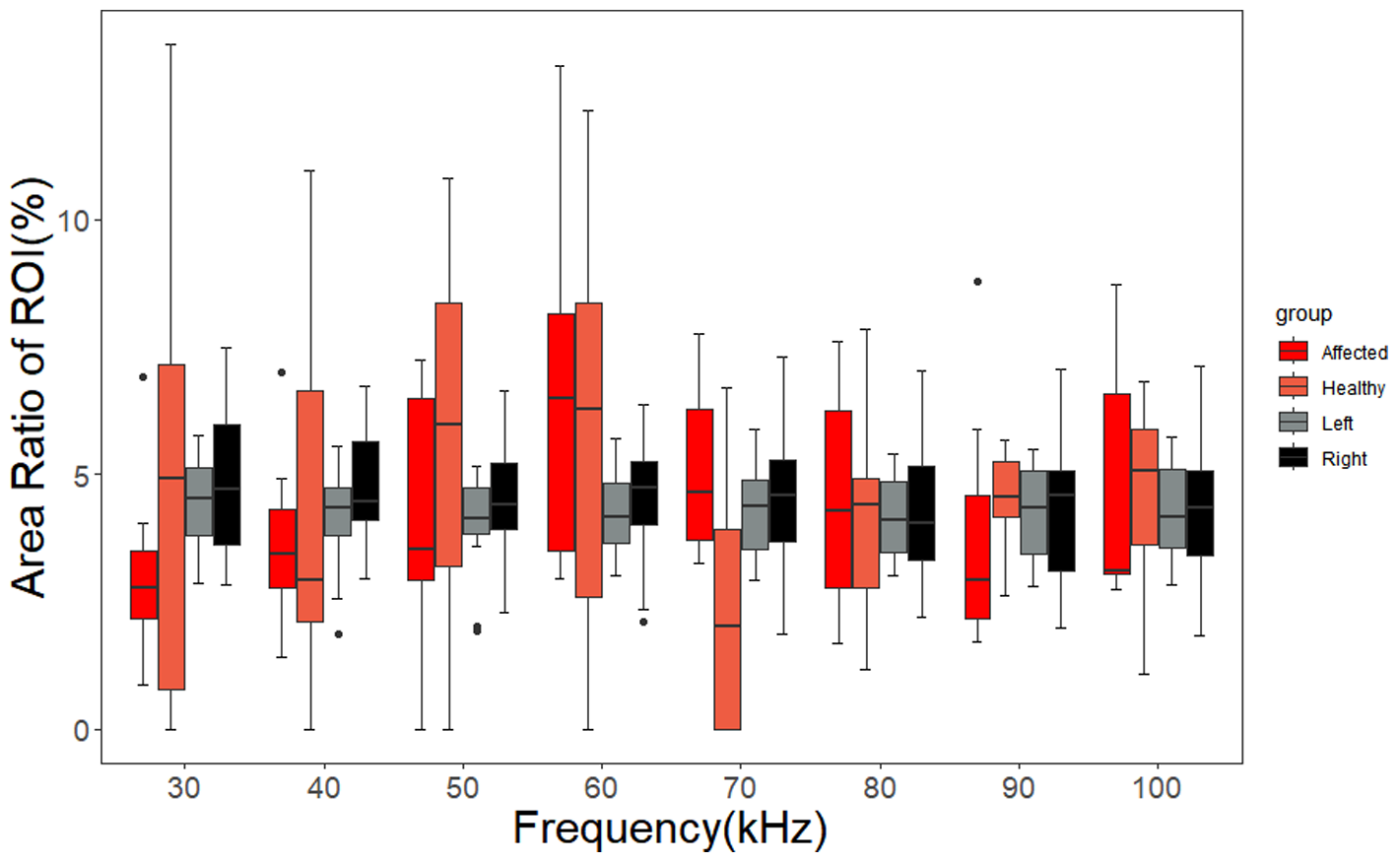
Area ratio of the ROIs of the healthy volunteers and the patients with brain diseases. The ARRs of the affected group and the healthy group correspond to the ROIs on the affected sides and normal sides of the patients, respectively. The left group and the right group of ARRs correspond to the ROIs on the left sides and right sides of the healthy volunteers, respectively. Black dots are outliers.

The area ratio of the ROIs on the left and right sides of cerebral MFEIT images of healthy volunteers and the area ratio of ROIs on the affected and normal sides of cerebral MFEIT images of patients with brain diseases at 8 frequency points in the range of 30 kHz-100 kHz are shown in Figure 7. There was no significant difference in the area ratio of the ROIs between the left and right sides of cerebral MFEIT images of healthy volunteers, and there was also no significant difference in the area ratio of the ROIs between the affected and normal sides of cerebral MFEIT images of patients with brain diseases (*p* > 0.05). All box plots report median (black line in the middle), quartiles (boxes), and data ranges, excluding outliers (black dots).

**Figure 8:**
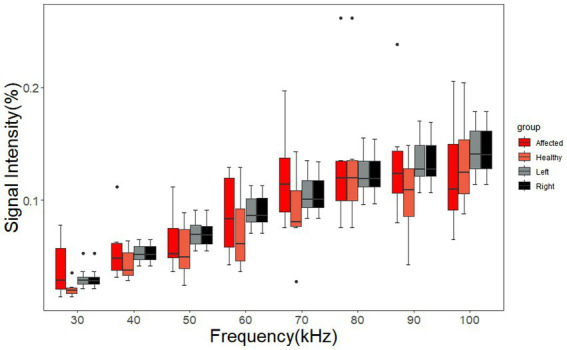
Signal intensity of the ROIs of the healthy volunteers and the patients with brain diseases. The affected group and the healthy group of SIs correspond to the ROIs on the affected sides and normal sides of the patients, respectively. The left group and the right group of SIs correspond to the ROIs on the left sides and right sides of the healthy volunteers, respectively. Black dots are outliers.

The signal intensity of the ROIs on the left and right sides of cerebral MFEIT images of the healthy volunteers and the signal intensity of the ROIs on the affected and normal sides of cerebral MFEIT images of patients with brain diseases at eight frequency points in the range of 30 kHz-100 kHz are shown in Figure 8, with no significant difference in ROI signal intensity between the two sides of healthy volunteers (*p* > 0.05) and no significant difference in ROI signal intensity between the affected side and the normal side of the brain of patients with brain diseases (*p* > 0.05).

### 3.3. Analysis of the incidence of positive events

**Figure 9:**
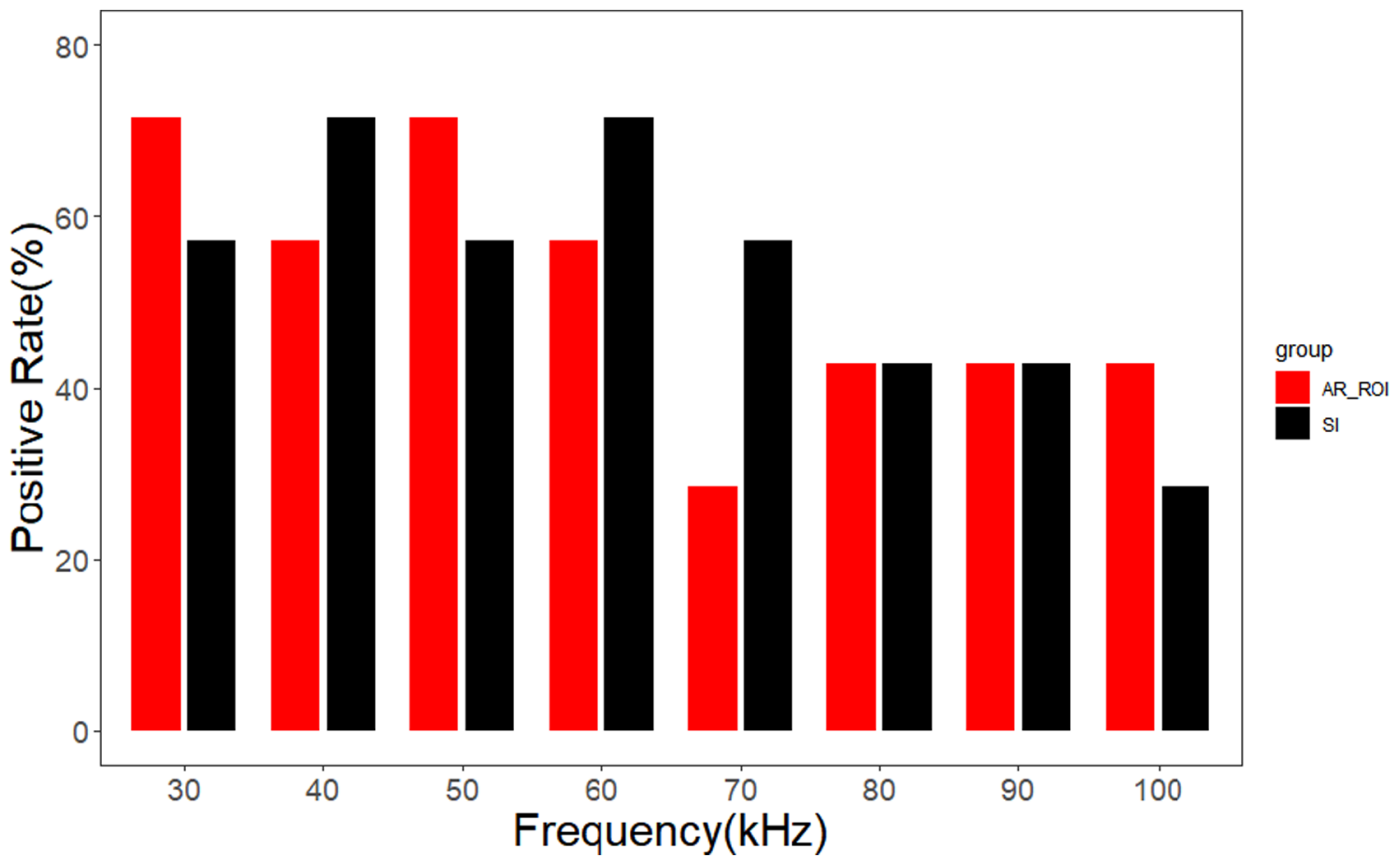
Positive rates of AR_ROI and SI.

As shown in Figure 9, for AR_ROI, the positive rate is higher than 50% in the range of 30 kHz – 60 kHz. In terms of signal intensity (SI), the positive rate is higher than 50% in the range of 30 kHz – 70 kHz. Taking the intersection of two ranges indicates that the incidence of positive events related to both AR_ROI and SI is higher than 50% in the range of 30 – 60 kHz. In this frequency range, the area or signal intensity of the ROI on the affected side of patients with unilateral brain disease is lower than that on the healthy side, which makes it easy for positive events to be identified.

### 3.4. Analysis of the symmetry of MFEIT images

**Figure 10:**
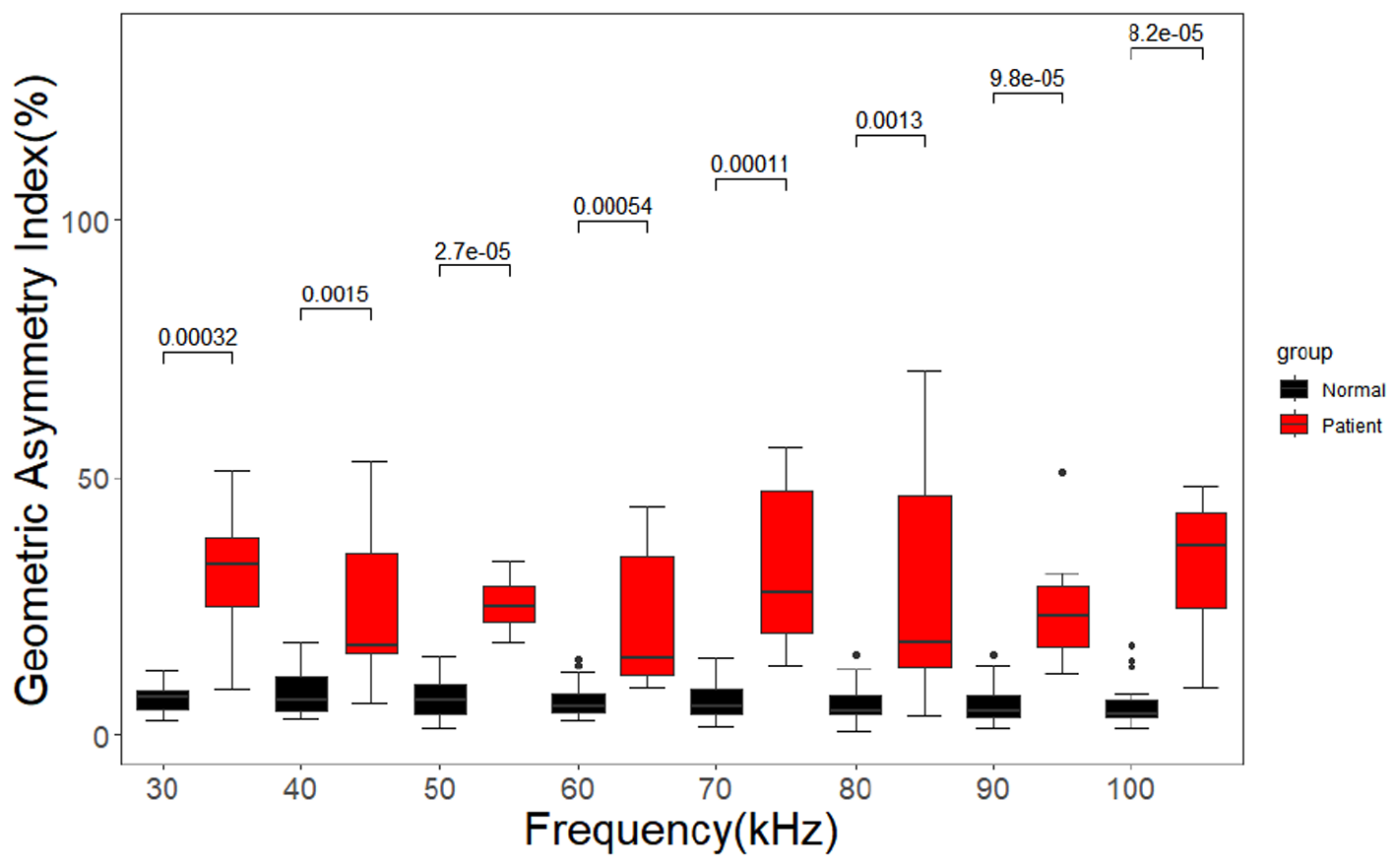
Geometric asymmetry index for the healthy volunteers and the patients with brain diseases. Black dots are outliers.

The geometric asymmetry index of the cerebral MFEIT images of healthy volunteers was significantly lower than that of patients with brain diseases (*p* < 0.05) after the Wilcoxon rank sum test at eight frequency points in the range of 30 –100 kHz as shown in Figure 10.

**Figure 11:**
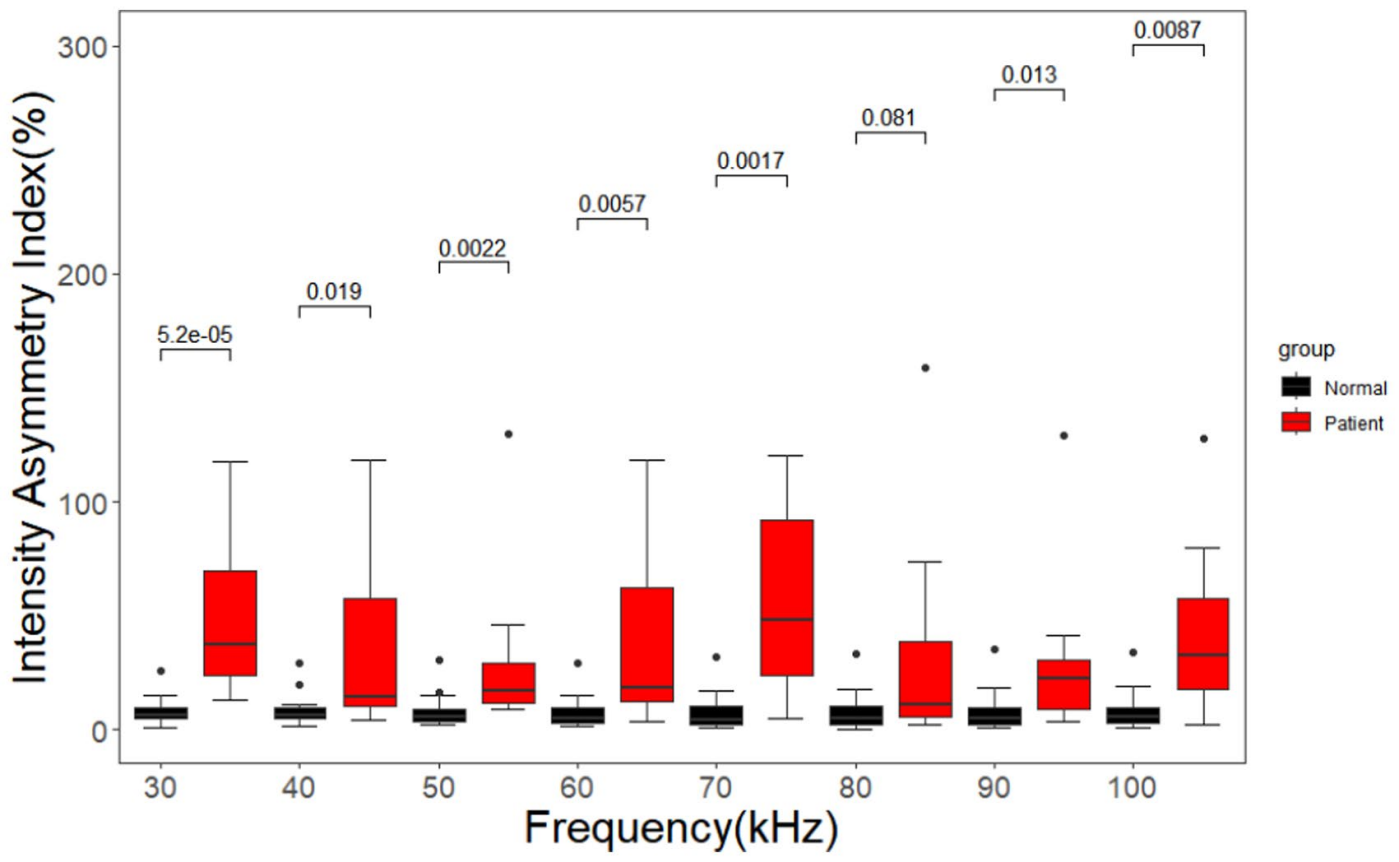
Asymmetry index of the signal intensity for healthy volunteers and patients with brain diseases. Black dots are outliers.

As shown in Figure 11, the signal intensity asymmetry index of the cerebral MFEIT images of healthy volunteers was significantly lower than that of the corresponding images of patients with brain diseases at the same 8 frequency points after the Wilcoxon rank sum test except at 80 kHz (*p* < 0.05).

## 4. Discussion

Brain diseases such as stroke and brain injury have high mortality and disability rates. Cerebral edema secondary to cerebral hemorrhage, cerebral ischemia and brain injury often leads to increased intracranial pressure, and high intracranial pressure can easily induce life-threatening conditions such as brain herniation (22). These intracranial conditions develop rapidly, so it is necessary to assess the patient’s intracranial condition in time to adjust the diagnosis and treatment strategy accordingly. EIT technology is advantageous in that it is noninvasive, harmless and allows continuous measurement of brain parameters. Based on the differences in the dielectric properties of different brain tissues, EIT data are selected for differential imaging in the frequency band where the resistivity spectrum of brain lesions differs from that of normal brain tissues, and the imaging results reflect the change in resistivity of brain tissues in each region with respect to frequency (20). The advantage of frequency-differential EIT over temporal differential EIT is that the former can reflect intracranial abnormalities in a one-time imaging manner without a reference EIT data corresponding to the time before the onset of brain diseases, which is helpful to obtain the patient’s intracranial condition in time. The electrical impedance group of UCL University (18, 23–25) and the electrical impedance group of the Fourth Military Medical University in China (26, 27) have conducted in depth studies of cerebral MFEIT, and the research strategy of selecting frequency bands of fdEIT based on the dielectric properties of biological tissues has been proven successful through physical and animal model studies (17). However, studies on the characteristics of fdEIT images and the differences in those characteristics between healthy subjects and patients with brain diseases have not been reported. For this reason, we explored the use of fdEIT imaging with measured cerebral MFEIT data from healthy volunteers and patients with brain diseases in the range of commonly measured EIT frequencies and investigated the characteristics and differences in imaging results between the two groups of subjects.

**Figure 12:**
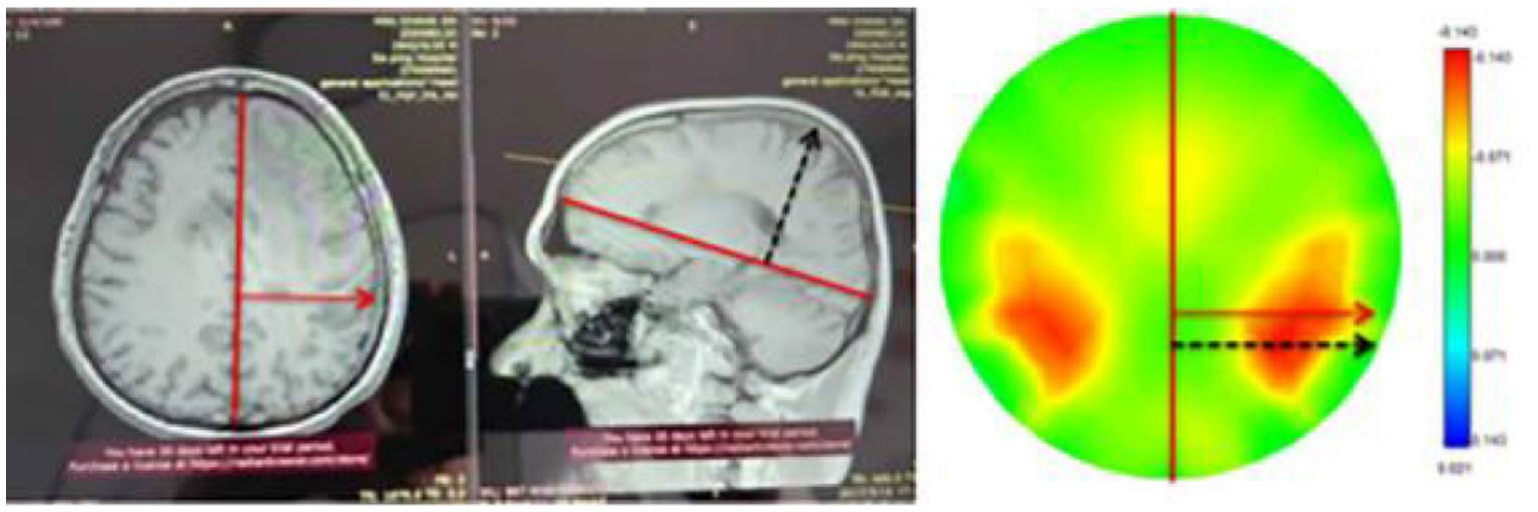
Maximum horizontal width and maximum thickness markers of the human brain and their identification on EIT images.

In this study, we performed MFEIT imaging on 16 healthy volunteers and 8 patients with brain diseases. From the MFEIT images of the human brain, the images of the healthy subjects are approximately left–right symmetric, which is consistent with the left–right approximate symmetry of the variations in resistivity of the normal brain tissues on both sides, and two stronger resistivity distribution regions approximately symmetric about the left–right symmetry axis can be observed within the posterior regions of the MFEIT images of the healthy human brain. This is similar to the results of MIT imaging of the healthy human brain reported by a Russian research group (28), see Figure 7 in the literature. It is clear from the analysis of the anatomical images of the human brain in the coronal and sagittal planes that in the posterior part of the temporal lobe, the horizontal width and the lead thickness of the brain tissue will reach a maximum, as shown in Figure 12 below, with the horizontal width maximum marked by the red arrow and the lead thickness maximum marked by the black dashed line. The horizontal width maximum and the lead thickness maximum are marked on the EIT image of one healthy volunteer according to the same geometric scale, and the two marking lines fall just in the area of stronger resistivity change on the EIT image, as shown in the right part of Figure 12. Thus, the larger spatial volume distribution of brain tissue in the posterior temporal lobe may be the reason for the two approximately left–right symmetrical distributions of stronger resistivity changes in the posterior part on the EIT images of the healthy human brain. On the contrary, the reconstructed images of the 8 patients were not as symmetrical as that of the healthy subjects. We selected a CT or MRI slice with the largest lesion size each for one patient to display the patient’s intracranial condition, even if the lesion is not in the electrode plane of EIT. The reason for this choice is that EIT does not just display resistivity distribution of the plane where the electrodes are located. It displays the projection of the distribution of electrical resistivity changes in the three-dimensional space of the measured head on the 2D plane. Under the current electrode configuration, even if the lesion is not on the electrode plane, it will still affect the distribution of electric fields applied to the measured head by EIT and can still be reconstructed by EIT.

As seen in the results of this study, the GAI and IAI of cerebral MFEIT images of the healthy subjects were significantly lower than those of the patients with brain diseases at the frequency range of 30 kHz-100 kHz, suggesting that the symmetry of MFEIT images in this frequency band could potentially be used for the rapid determination of abnormal intracranial conditions. Further analysis of the incidence of positive events in cerebral MFEIT images of the patients with brain diseases (especially with cerebral hemorrhage) shows that the resistivity change of the lesion region with the frequency was weaker than that of normal brain tissue in the contralateral region that was near-symmetrical to the lesion in the range of 30 kHz - 60 kHz. The dielectric properties of stroke lesions and normal brain tissue were reported in the literature in 2016 (29) (see Figure 4 in the literature). The amount of variation in the real part of the electrical impedance of each tissue with frequency in descending order in the range of 1 kHz-100 kHz is brain white matter > ischemic brain tissue > brain gray matter >hemorrhagic tissue. In the framework of the current fdEIT algorithm, whether the biological tissue in a certain cerebral region is imaged depends on the comparison of the impedance change with the frequency of the biological tissue in the region with that in the adjacent region: when there is a large change in impedance with the frequency of a certain tissue, the image of this tissue will be reconstructed with a larger area or a stronger signal intensity. In the patients with an intracranial hematoma in this study, the rate of electrical impedance change with the frequency of normal brain tissue was higher than that of the hemorrhagic lesion in the selected frequency band for fdEIT, so a stronger resistivity change distribution was presented in the contralateral area of the hemorrhagic lesion. It is known from the electrical impedance spectrum of biological tissues that the resistivity variation with frequency of healthy brain tissue is higher than that of brain lesions in the frequency range of EIT imaging in this study, the fdEIT image of the patient (e.g., Figure 6, row 4) showed a higher resistivity change on normal brain tissue in the contralateral region that was near-symmetrical to the lesion than that on the lesion side, which was different from the image performance on one side of a healthy volunteer. The rate of electrical impedance change with the frequency of biological tissues in the brain is a key factor in determining whether a certain tissue can be reflected by the fdEIT algorithm used in this study. Biological tissues with a larger rate of electrical impedance change with frequency will be reflected by the fdEIT in preference to the tissues with a smaller rate, presenting reconstructed targets in their corresponding locations of the image with a larger area or stronger intensity. The above experimental results demonstrate the feasibility of the current MFEIT for detecting intracranial abnormalities.

In addition, according to the dielectric properties of rabbit brain tissues and stroke tissues reported in the literature, the amount of resistivity variation with frequency is greater in ischemic tissue than in normal brain tissue and blood in the lower frequency band (DC-500 Hz) (30), while in the higher frequency band (200 kHz-1 MHz), the amount of resistivity variation with frequency is greater in blood than in normal brain tissues and ischemic brain tissue (29), Therefore, in the future, this study will broaden the measurement frequency range of the MFEIT system to allow a wider range of MFEIT measurements and imaging covering the above frequencies, thus enabling MFEIT to detect cerebral ischemia in the low frequency band and cerebral hemorrhage in the high frequency band and to show higher resistivity changes at the location corresponding to the lesions. This approach will allow a more visual representation of the presence and development of intracranial abnormalities.

In the observation of cerebral MFEIT images of each patient, we found that there was a stronger resistivity change area on the normal side of the patient and the area approximately symmetrical with the patient’s lesion about the axis of symmetry of the coronal plane of the brain. However, statistical analysis did not find a difference in the ROI area or ROI signal intensity between the affected sides and the normal sides of the patients. The possible reason is that the difference between normal brain tissue and lesions in the reconstruction resistivity changes on the fdEIT images of these frequencies is small. Although the number of patients in the present study is small, in future research, more patients with brain diseases can be recruited to participate in cerebral MFEIT imaging experiments to further support the rule mentioned above. There has been some progress in image feature recognition in AI technology (31, 32). Zhang Tao summarized the application progress of depth learning in EIT image reconstruction from three aspects: single network reconstruction, depth learning combined with traditional algorithm reconstruction, and multi-network hybrid reconstruction (33). In general, AI technology provides a new method to improve the performance of EIT image reconstruction. In the future, to ensure that sufficient cerebral MFEIT images of patients with brain diseases are available, researchers can use artificial intelligence technology such as an artificial neural network (ANN) or support vector machine (SVM) to identify the affected side and the healthy side of the patient’s brain based on MFEIT images and estimate the location of the lesions.

**Figure 13:**
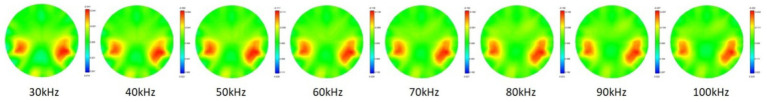
Cerebral MFEIT imaging results of a suspected brain disease patient.

The method of detecting intracranial abnormalities based on the symmetry of MFEIT images proposed in this study may have two benefits. The first benefit is that healthy people in the community or grassroots clinics can be screened to identify potential brain diseases at an early stage for effective measures to be taken that may reduce the occurrence of brain diseases. In this regard, we have encountered a typical case. A staff member of Daping Hospital of Army Medical University (male, 44 years old) asked us for help during the period we carried out cerebral MFEIT data collection on healthy volunteers. He reported that he had frequent pain in the right side of the brain and wanted to have a look at the brain through MFEIT. We conducted a standard MFEIT data acquisition procedure for him according to the experimental protocol in this paper and found that the MFEIT images of his brain were asymmetric compared with the normal subjects, and the area of the ROI reconstructed on the right side of each image was smaller than that on the left side (as shown in Figure 13). Based on this result, we estimated that there might be abnormalities in the right brain of this person. We suggested further brain imaging examination. Brain CT showed that the subject had a strip of high-density shadow on the right side of the suprasellar area. Cerebral angiography (CTA) showed that there were abnormalities on the right side of the subject’s brain: tortuous C7 segment of the right internal carotid artery and right embryonic posterior cerebral artery. After inquiry, the subject was found to be addicted to alcohol and prone to excitement. We suggest that he reduce alcohol consumption, control emotions, and monitor blood pressure to prevent brain diseases such as cerebral hemorrhage that may occur in the later stage. The second benefit is that MFEIT can be used to continuously collect data during the monitoring of critical neurosurgical patients and evaluate the intracranial abnormalities of such patients in real time based on changes in the symmetry of the obtained MFEIT images, thereby possibly preventing the occurrence of secondary cerebral hemorrhage, cerebral hernia and other emergencies.

There are several limitations of this study that need to be noted. First, this study was an exploratory study, so the number of patients recruited was relatively small, and second, the patients recruited in this study were at different stages of morbidity. In future studies, more precise detection of intracranial conditions, such as accurate localization of lesions and identification of lesion types, will require optimization of the clinical protocol and grouping of subjects according to different types of brain diseases, different stages of the diseases (i.e., different times from the onset of the diseases), and different lesion locations. Third, the EIT imaging is two-dimensional, and a two-dimensional EIT image reflects the projection of the three-dimensional resistivity changes of the measured object on the two-dimensional plane, so the spatial resolution of 2D EIT is difficult to be defined. Therefore, the spatial resolution of 2D EIT image is not evaluated at present. In future research, we will use a 3D EIT with higher spatial resolution to reflect intracranial abnormalities of the measured object. And the spatial resolution of 3D EIT image will be evaluated accurately. Four, the main purpose of the study is to explore detection of intracranial abnormalities based on the difference in the symmetry of multi-frequency EIT images between healthy volunteers and patients with brain diseases, so we have not yet evaluated the performance of fdEIT in detecting lesion size. However, in our previous tdEIT imaging research, it was found that the similar EIT system could detect resistivity disturbances that account for 0.35% of the total volume of the imaged target (34). In further studies, after accumulating a large amount of patient data, we may be able to establish the relationship between the size of reconstruction targets on fdEIT images and the lesion size evaluated by CT of MRI. Then fdEIT will be a useful tool to detect intracranial conditions.

## 5. Conclusion

In summary, this was the first study to analyze the image characteristics of the fdEIT imaging results of healthy subjects and patients with brain diseases. The results of the study showed that the asymmetry of the fdEIT images was significantly higher in patients with brain disease than in healthy subjects. This finding provides evidence for further exploration of the multifrequency EIT method in the clinical detection of intracranial abnormalities.

## Data availability statement

The raw data supporting the conclusions of this article will be made available by the authors, without undue reservation.

## Ethics statement

The studies involving human participants were reviewed and approved by the Ethics Committee of the Army Medical Center of PLA. The patients/participants provided their written informed consent to participate in this study. Written informed consent was obtained from the individual(s) for the publication of any potentially identifiable images or data included in this article.

## Author contributions

JieM performed the overall design of the study and collected MFEIT data from the heads of healthy volunteers. JG calibrated and analyzed the experimental data. YL analyzed the features of MFEIT images. ZW, YD, and JianM collected cerebral MFEIT data and imaging data from patients with brain diseases. YZ and GW implemented and optimized the imaging algorithm of the EIT system. LY designed and guided the clinical experiments. XS provided guidance on EIT data analysis. All authors contributed to the article and approved the submitted version.

## Funding

This work was supported by the Basic Strengthen Project under Grant 2020-JCJQ-ZD-254-05, by the National Key R&D Plan Project under Grant 2022YFC2404803, by the Project of Talent Innovation Ability Training Program of the Army Medical Center of PLA under Grant 2019CXJSC021, and by the Special Project for Improving the Scientific and Technological Innovation Capability of the Army Military Medical University under Grant 2019XYY26.

## Conflict of interest

YZ and GW were employed by Hangzhou Utron Technology Co., Ltd.

The remaining authors declare that the research was conducted in the absence of any commercial or financial relationships that could be construed as a potential conflict of interest.

## Publisher’s note

All claims expressed in this article are solely those of the authors and do not necessarily represent those of their affiliated organizations, or those of the publisher, the editors and the reviewers. Any product that may be evaluated in this article, or claim that may be made by its manufacturer, is not guaranteed or endorsed by the publisher.
