# Supplementary figures and images for: Exploratory study of a multifrequency EIT-based method for detecting intracranial abnormalities
Source: Front Neurol. 2023 Aug 11;14:1210991. doi: 10.3389/fneur.2023.1210991 (PMC10457004; doi:10.3389/fneur.2023.1210991)

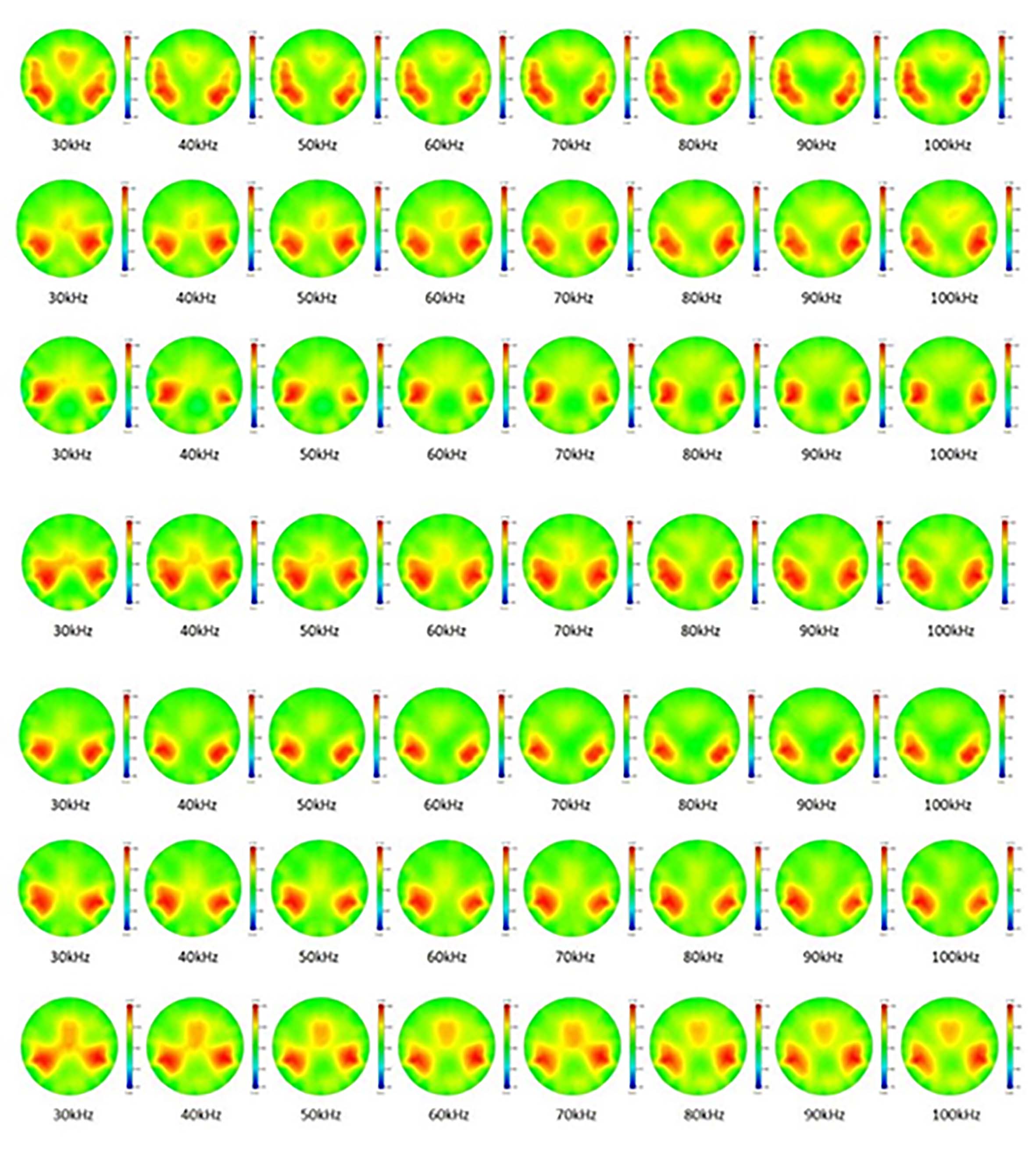

Supplement: Supplementary file 1 [file Image_1.JPEG]

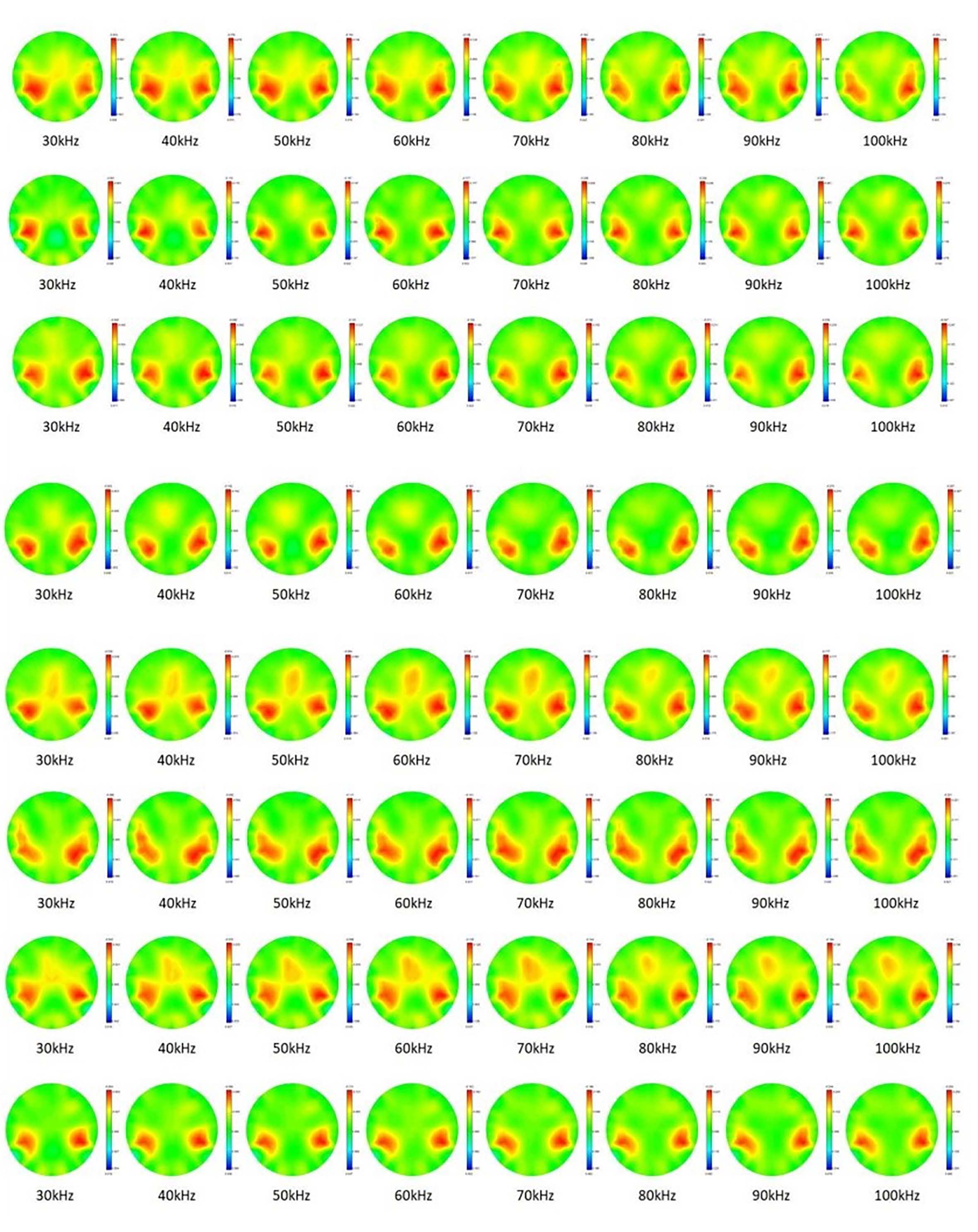

Supplement: Supplementary file 2 [file Image_2.JPEG]
